# Transcriptome Analysis Reveals Long Intergenic Noncoding RNAs Contributed to Growth and Meat Quality Differences between Yorkshire and Wannanhua Pig

**DOI:** 10.3390/genes8080203

**Published:** 2017-08-18

**Authors:** Cheng Zou, Sha Li, Lulu Deng, Yang Guan, Dake Chen, Xiongkun Yuan, Tianrui Xia, Xinglin He, Yawei Shan, Changchun Li

**Affiliations:** Key Lab of Agriculture Animal Genetics, Breeding, and Reproduction of Ministry of Education, College of Animal Science and Technology, Huazhong Agricultural University, Wuhan 430070, China; zzcy873704025@163.com (C.Z.); lovguyls@163.com (S.L.); 15171492670@163.com (L.D.); gy963967268@163.com (Y.G.); dakelchan@163.com (D.C.); yuanxiongkun@126.com (X.Y.); phillislis@163.com (T.X.); xinglinhe@yeah.net (X.H.); shansyw799@163.com (Y.S.)

**Keywords:** lincRNAs, Yorkshire pig, Wannanhua pig, muscle growth, meat quality

## Abstract

There are major differences between Yorkshire (lean-type) and Wannanhua pig (fat-type) in terms of growth performance and meat quality. Long intergenic noncoding RNAs (lincRNAs) are a class of regulators that are involved in numerous biological processes and widely identified in many species. However, the role of lincRNAs in pig is largely unknown, and the mechanisms by which they affect growth and meat quality are elusive. In this study, we used published data to identify 759 lincRNAs in porcine longissimus dorsi muscle. These putative lincRNAs shared many features with mammalian lincRNAs, such as shorter length and fewer exons. Gene ontology and pathway analysis indicated that many potential target genes (PTGs) of lincRNAs were involved in muscle growth-related and meat quality-related biological processes. Moreover, we constructed a co-expression network between differentially expressed lincRNAs (DELs) and their PTGs, and found a potential mechanism that most DELs can use to upregulate their PTGs, which may finally contribute to the growth and meat quality differences between the two breeds through an unknown manner. This work details some lincRNAs and their PTGs related to muscle growth or meat quality, and facilitates future research on the roles of lincRNAs in these two types of pig, as well as molecular-assisted breeding for pig.

## 1. Introduction

In past decades, Western commercial pigs have been intensively selected for higher growth and lean meat content; however, this process is believed to contribute to the retrogradation of meat quality [1]. As a typical lean-type Western breed, the Yorkshire (YY) pig is now widely used for commercial production [2]. Compared with Western commercial breeds, although Chinese indigenous pig breeds, such as the Wannanhua (WH) pig (fatty), exhibit a lower growth rate and lean rate, the WH pig has a higher intramuscular fat content and superior meat quality. Therefore, these two breeds can serve as an ideal comparison for studying differences in growth performance and meat quality between Western commercial pigs and Chinese indigenous pigs [2].

Skeletal muscle is the most abundant and major metabolic tissue in pig [3]. As a typical kind of skeletal muscle, longissimus dorsi muscle comprises different fibre types that differ in terms of metabolism (oxidative and glycolytic) and biochemical characteristics (glycogen and lipid content), and can result in differences in meat quality [4,5,6]. In previous studies, Liu et al. and Li et al. explained the phenotypic differences (mainly growth performance and meat quality) between Yorkshire and Wannanhua pigs in the perspective of protein-coding genes and microRNAs [2,7]. Moreover, many genes and microRNAs were found to be involved in growth-related or lipid metabolism-related processes in other studies. For example, Zhang et al. proved that myogenic regulatory factors (MRFs) were critical for successful myogenesis [8], and Yue et al. demonstrated that adipocyte determination and differentiation factor-1 (ADD1) expression was related to adipocyte differentiation in pigs [9]. Besides, Feng et al. found that *microRNA-214* can influence skeletal muscle development by regulating embryonic myogenesis [10], and Li et al. revealed that *microRNA-103* had an impact on porcine preadipocyte differentiation through the putative target gene *RAI14* [11]. However, research about the effect of long intergenic noncoding RNAs (lincRNAs) on muscle growth and meat quality has been rare.

LincRNAs are a class of intergenic transcripts that are longer than 200 bp in length and barely have protein-coding capacity. In recent years, studies have identified numerous lincRNAs, and proven that some of them play important role in various biological processes, such as gene regulation [12,13], genomic imprinting [14], and skeletal muscle development [15]. However, many lincRNAs in pigs remain unidentified compared with humans and mice [16,17,18]. The relationship between lincRNAs and their potential target genes (PTGs) are still unclear, and lincRNAs that affect muscle growth and meat quality in pig are yet to be elucidated.

In this study, we performed transcriptome assembly of longissimus dorsi muscle transcriptomes of Yorkshire and Wannanhua pigs using published data from a previous study [2]. We identified a total of 759 putative lincRNAs and characterized the basic features of these lincRNAs. Based on the expression information, we detected some differentially expressed lincRNAs between the two breeds. Gene ontology and pathways analysis were carried out on the PTGs of lincRNAs and revealed that some of these PTGs significantly participated in some muscle growth-related and meat quality-related biological processes. Combined with the differential expression analysis results of mRNA, we found most of differentially expressed lincRNAs (DELs) can positively regulate their PTG expression. This study paves the way for future studies exploring the functional roles of specific lincRNAs that may contribute to the growth and meat quality differences between Western commercial pigs and Chinese indigenous breeds.

## 2. Materials and Methods

### 2.1. Ethics Statement and Data Acquisition

In this study, the sows used for RNA-seq were reared under the same environmental and nutritional conditions [2]. All samples were taken from the same part of the longissimus dorsi muscle, as described by Li et al. [2]. Experiments were performed according to the Regulations for the Administration of Affairs to Concerning Experimental Animals and approved by the Animal Research Committee of Anhui Academy of Agriculture Sciences and Anhui Agricultural University [2]. All RNA-seq data (YY, three samples; WH, three samples) used were downloaded from the NCBI Gene Expression Omnibus (GEO) databases with the accession number offered by Li et al. (Table 1) [2].

### 2.2. Publicly Available Annotations

In this study, the pig gene annotations were downloaded from Ensembl database [19]. The pig lincRNA annotations were derived from http://res.xaut.edu.cn/aldb/download.jsp [20,21]. Moreover, the nonredundant reference sequence (RefSeq) non-redundant (NR) database was downloaded from National Center for Biotechnology Information (NCBI) [22], and the human and mouse lincRNAs references were both downloaded from Ensembl database ([23] for the human lincRNAs and [24] for the mouse lincRNAs).

### 2.3. RNA-Seq Reads Mapping and Transcriptome Assembly

The raw reads were cleaned by filtering the adapter and low-quality reads by Trimmomatic (v0.36) [25]. First, the adaptors were removed; then, low quality reads (the number of mismatch >2 in a read) were removed, and the reads in which the average quality of four continuous bases were <15 were discarded. The clean reads were mapped to the pig reference genome (*Sus scrofa* 10.2, ftp://ftp.ensembl.org/pub/release-87/fasta/sus_scrofa/dna/) by Tophat v2.0.14 [26] with default parameters. Then, the mapped reads were assembled through Cufflinks v2.2.1 [27] with default parameters. Meanwhile, we set the “-g” option of Cufflinks for novel transcript assembly. Six assembled transcript files (GTF format) of two groups were then merged into a nonredundant transcriptome using the Cuffmerge utility provided by the Cufflinks package. The nonredundant transcriptome was then filtered according to the lincRNA detection pipeline.

### 2.4. Pipeline for lincRNA Identification

We used the following steps to identify lincRNAs from the nonredundant transcriptome: (1) only transcripts with ‘u’ category categorized by Cuffmerge, which indicated intergenic transcripts, were retained; (2) transcripts with a single exon or less than 200 bp in length were filtered out; (3) the Coding Potential Calculator (CPC) [28] tool was used to calculate the coding potential of transcripts in both strands, and transcripts with a CPC value >0 in any strands were removed; (4) the remaining transcripts that contained any known protein-coding domain were discarded. To accomplish this, we translated transcripts sequence into six possible protein sequences by Transeq and used HMMER to exclude those transcripts whose corresponding protein sequences had a significant hit in the Pfam database (*E*-value < 1 × 10^−5^) [29]; (5) any transcripts with similarity to known proteins against the NCBINR and UniRef90 database with an *E*-value < 1 × 10^–5^ were filtered out using the BLASTX [30] program; (6) any transcripts with a fragments per kilobase of transcript per million mapped reads (FPKM) score lower than 0.5 in all samples were discarded.

### 2.5. Differentially Expressed lincRNAs and mRNA Analysis

We used the Cuffdiff utility provided by the Cufflinks package to conduct differential expression tests between two breeds. The fold changes were calculated via log_2_ (FPKM_YY/FPKM_WH) (FPKM_YY: FPKM of group YY; FPKM_WH: FPKM of group WH). A transcript will be identified as differentially expressed between two breeds if the false discovery rate (FDR)-adjusted *p*-value is less than 0.05 [31].

### 2.6. Prediction of PTGs of lincRNAs

We predicted the PTGs of lincRNAs in two ways. For PTGs that were potentially regulated by lincRNAs in *cis*, we defined a lincRNA PTG as protein-coding genes that were transcribed nearby (<10 kb) lincRNAs [32,33], and we identified this kind of PTGs by BEDTools 2.17.0 [34]. For PTGs that were potentially regulated by lincRNAs in trans, we referred to Liao’s study and constructed a lincRNA–mRNA co-expression network based on the Pearson correlation coefficient (*r*) between each pair of lincRNA and protein-coding gene. We regarded a protein-coding gene as a PTG of lincRNA only when the *r* between them ≥0.95, and the FDR-adjusted *p*-value ≤ 0.05 [31,35]. To minimise our false positive, we selected protein-coding genes and lincRNAs that had detectable expression in all six samples.

### 2.7. Gene Ontology and Pathway Analysis

We performed Database for Annotation, Visualization and Integrated Discovery (DAVID) analysis by running queries for each PTG against the DAVID database [36]. Because of the limited annotation of the porcine genome, all of the PTGs were firstly converted into human homologous genes using BIOMART from Ensembl [37].

### 2.8. Correlation Validation between lincRNAs and Their PTGs

We performed validation of the relationship between lincRNA and their PTG in another longissimus dorsi muscle dataset, which contained 18 samples (NCBI GEO database, accession GSE65983) [38]. First, we calculated the expression of lincRNAs and their PTGs by HTSeq [39]. Then, we carried out regression analysis between lincRNAs and their PTGs by homemade R script.

## 3. Results

### 3.1. Transcripts Assembly and lincRNAs Identification

**Figure 1:**
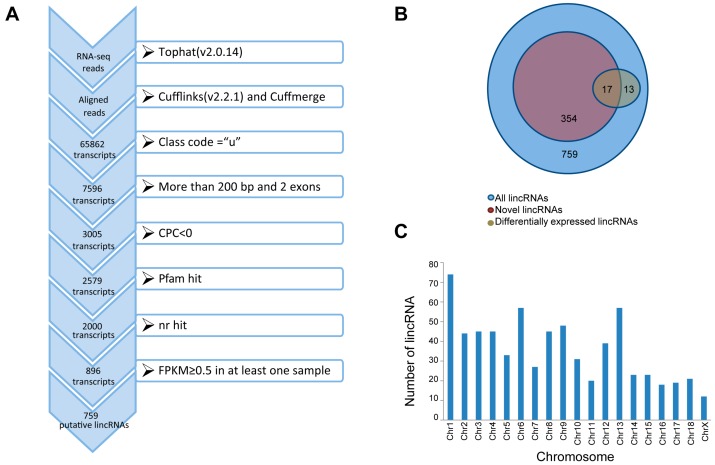
(**A**) Overview of the identification pipeline for putative long intergenic noncoding RNAs (lincRNAs) in this study; (**B**) Number of different kinds of lincRNAs; (**C**) The chromosome distribution of lincRNAs. CPC: Coding Potential Calculator; nr: non-redundant; FPKM: fragments per kilobase of transcript per million mapped reads.

To identify the lincRNAs in longissimus dorsi muscle that contribute to the growth and meat quality differences between Western commercial and Chinese indigenous pigs, we used RNA-seq data from a previously published study involving two types of pig: Yorkshire and Wannanhua [2]. After removing the adaptor sequences and discarding low-quality reads, about 283.3 of 369.0 million clean reads were mapped to the whole genome of *Sus scrofa* (10.2) (Table 1). Then, we reconstructed the transcriptome for each sample through Cufflinks, and all of the transcripts were pooled into a unique merged transcript set through Cuffmerge [40]. We obtained a total of 65,862 transcripts, of which 7596 were intergenic transcripts. We identified lincRNAs from the 7596 transcripts according to the illustration shown in Figure 1A. Finally, we obtained 759 putative lincRNAs encoded by 542 gene loci, and 354 of the 759 lincRNAs have no overlap with currently annotated coding or noncoding transcripts (Figure 1B; Table S1). These putative lincRNAs were distributed in all of the chromosomes except the Y chromosome (Figure 1C).

### 3.2. Characterization of Identified lincRNAs

**Figure 2:**
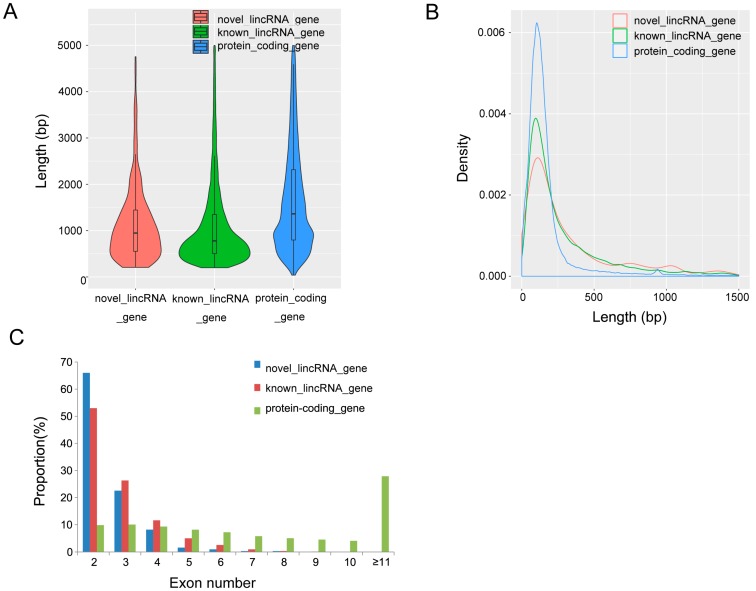
Comparisons of transcript length, exon length, and exon number. (**A**) Comparisons of transcript length. Novel lincRNA genes show shorter average transcripts length (1226 bp) than that of the known lincRNA genes (1362 bp) and the protein-coding genes (1983 bp), the curve indicates density distribution; (**B**) Comparisons of exon length. Novel lincRNA genes show longer mean exon length (466 bp) than that of the known lincRNA genes (451 bp) and protein-coding genes (228 bp); (**C**) Comparisons of exon number. Novel lincRNA genes trend to have fewer exons (2.6) than that of the known lincRNA genes (2.8) and protein-coding genes (8.7).

Based on the reconstructed transcriptome, we analyzed the features of novel lincRNAs and compared these features with those of protein-coding genes and known lincRNAs. There are 26,712 protein-coding transcripts corresponding to 21,607 genes in the pig annotation in the Ensembl database, and 12,103 known lincRNA transcripts corresponding to 7381 lincRNA genes in the pig lincRNA annotation in the domestic animal lincRNA database (ALDB) [24]. We found the average transcripts length of novel lincRNA genes in our study was 1226 bp, which is shorter than that of the known lincRNA genes (1362 bp) and protein-coding genes (1983 bp) (Figure 2A). Meanwhile, the average exon length of lincRNA genes was 466 bp, which is longer than that of the known lincRNA genes (451 bp) and protein-coding genes (228 bp) (Figure 2B). Furthermore, we found that the average exon number of novel lincRNA genes (2.6) was similar to that of the known lincRNA genes (2.8), but fewer than that of the protein-coding genes (8.7) (Figure 2C). LincRNAs lack protein-coding capacity, so we compared the expression level of the 759 lincRNAs in our study with that of protein-coding genes in two groups. Our results showed that lincRNAs have a lower average expression level than that of protein-coding genes in both groups (4.7 FPKM vs. 16.0 FPKM in the WH group; 6.5 FPKM vs. 17.1 FPKM in the YY group). These features of lincRNAs (shorter transcript length, longer exon length, fewer exon number, and lower expression level) compared with protein-coding genes are in agreement with the results of other studies [41,42,43] .

### 3.3. Differential Expression Analysis of lincRNAs and mRNA

**Figure 3:**
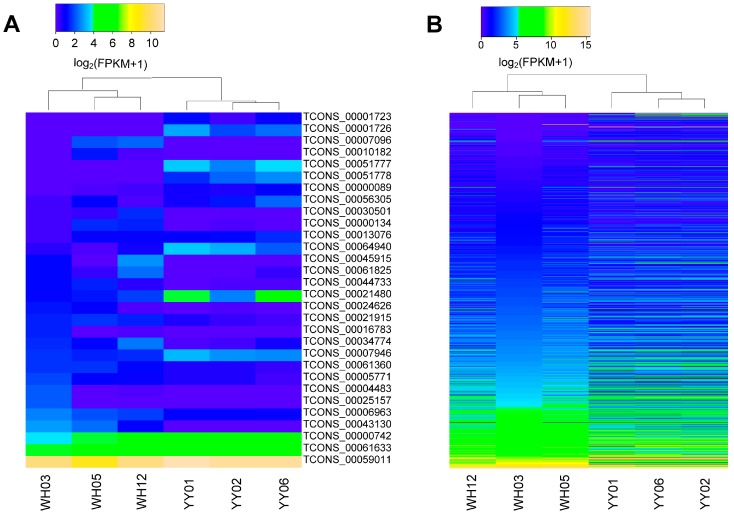
Expression of lincRNAs in two groups. Shown are heat maps of the log_10_ transformed FPKM + 1 expression values for differentially expressed lincRNAs and mRNA. The density of the color scheme is calibrated to the log_10_ expression level, such that yellow refers to higher expression, while blue refers to lower expression. The bar code represents the color scale of the log_10_ (FPKM + 1). WH: the Wannanhua (WH) group; YY: the Yorkshire (YY) group. (**A**) 30 differentially expressed lincRNAs between the WH group and the YY group; (**B**) 926 differentially expressed mRNAs between the WH group and the YY group.

The transcript expression levels were normalized to FPKM values using Cufflinks. Using Cuffdiff, we conducted the differential expression analysis between the YY and WH samples for exploring the function of the lincRNAs. We detected a total of 30 DELs between the two breeds. In detail, 17 upregulated and 13 downregulated DELs in the WH group compared with the YY group (Figure 3A, Table S2). Moreover, we detected 926 differentially expressed protein-coding genes (DEGs), of which 454 of them were upregulated and 472 were downregulated in the WH group compared with the YY group (Figure 3B).

### 3.4. Prediction of Differentially Expressed lincRNA Target Gene

Many studies have demonstrated that lincRNAs can regulate gene expression as *cis* regulators or *trans* regulators [44,45]. We predicted the PTGs of lincRNAs in two ways (see Materials and Methods). In our study, we mainly focused on DELs in later analysis. For PTGs regulated by lincRNAs in *cis*, we obtained eight PTGs of seven DELs. However, these eight PTGs were not differentially expressed between the two groups. For PTGs regulated by lincRNAs in *trans*, we identified a total of 2747 PTGs corresponding to 16 DELs, and 352 of the 2747 PTGs were differentially expressed between two groups. The number of differentially expressed PTGs (DEPTGs) for each DEL varied greatly. For example, lincRNA TCONS_00061360 had 110 target genes, which is the maximum among these lincRNAs, followed by lincRNA TCONS_00044733 and TCONS_00021915 with 80 and 71 target genes, respectively; while some lincRNAs such as TCONS_00013076 had only three target genes (Table 2).

### 3.5. Gene Ontology and Pathway Analysis of PTGs and mRNA

**Figure 4:**
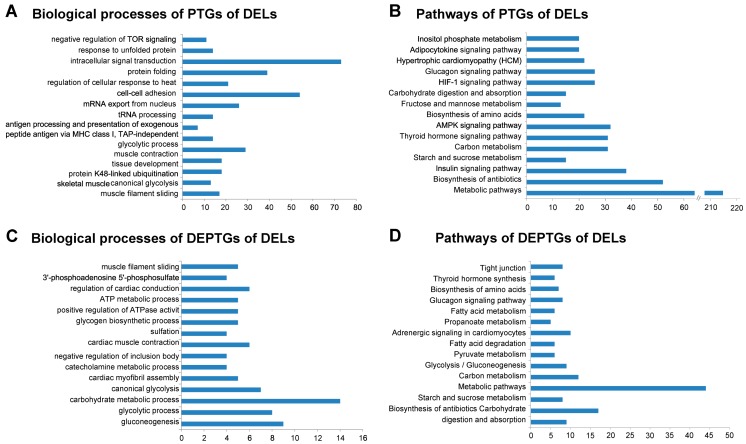
Gene ontology and pathway analysis of the potential target genes (PTGs) of differentially expressed lincRNAs (DELs). The x-axis indicates the number of genes, and the y-axis indicates different biological processes. (**A**) Biological processes of PTGs of DELs. (**B**) Pathways of PTGs of DELs. (**C**) Biological processes of (differentially expressed potential target genes) DEPTGs of DELs. (**D**) Pathways of DEPTGs of DELs.

In order to understand the functions and associated pathways of these PTGs of DELs, we performed DAVID analysis by running queries for each PTG against the DAVID database. The DAVID results revealed that 1002 of 2747 PTGs significantly participated in 132 biological processes. Some of these biological processes were muscle-related or lipid-metabolism-related, such as skeletal muscle tissue development, the glycolytic process, and fatty acid beta-oxidation (*p* < 0.05) (Figure 4A; Table S3). Besides, 534 PTGs were significantly involved in 50 pathways, including the biosynthesis of amino acids, and the adipocytokine signaling pathway (*p* < 0.05) (Figure 4B; Table S3). Furthermore, we also performed DAVID analysis of the 352 DEPTGs of DELs. The DAVID results revealed that 113 of 352 PTGs significantly participated in 65 biological processes, including the glycolytic process and fatty acid beta-oxidation using acyl-CoA dehydrogenase (*p* < 0.05) (Figure 4C; Table S4). In addition, 82 PTGs were significantly involved in 28 pathways, including glycolysis/gluconeogenesis and fatty acid degradation (*p* < 0.05) (Figure 4D, Table S4). Moreover, we also performed DAVID analysis on DEGs. The results of DAVID analysis on DEGs were in accordance with Li’s study (Table S5) [2].

### 3.6. Expression Regulation Analysis of DELs and Their Differentially Expressed Potential Target Genes

**Figure 5:**
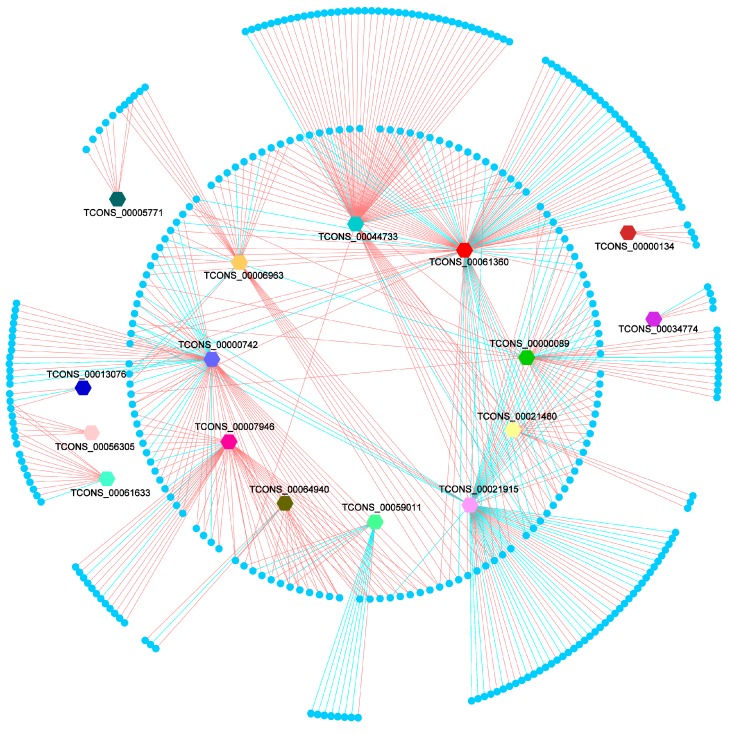
Co-expression network of DEPTGs and DELs. Bigger colored hexagons represent DELs; smaller circles represent DEPTGs; a red edge indicates DELs that upregulate DEPTGs; blue edge indicates DELs that downregulate DEPTGs; mRNAs’ names are not shown.

In order to explore the expression relationship between lincRNAs and PTGs, we analyzed the expression statues of DELs and their DEPTGs. Based on the expression level of the 352 PTGs and corresponding 16 DELs, we found that 14 of 16 DELs could upregulate the majority of their DEPTGs, and only 2 DELs (TCONS_00021915, TCONS_00059011) exhibited a contrary trend (Table 2; Figure 5). This result indicated that most DELs could promote the expression of the majority of their PTGs. Furthermore, we also found that 149 of 352 PTGs were regulated by more than one lincRNA, (Table S6; Figure 5), which implied that complicate regulating mechanisms exist between lincRNAs and their PTGs.

### 3.7. Validation of the Correlation between lincRNAs and Their Potential Target Genes

**Figure 6:**
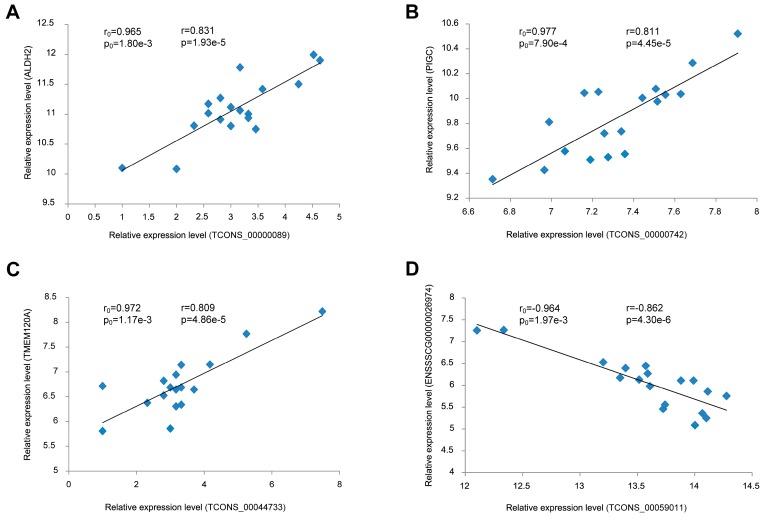
Linear regression of DEL and DEPTG expression. The *r*_0_ and *p*_0_ indicate the Pearson correlation coefficient and *p* value of each pair of DEL and DEPTG in six samples (three for the Wannanhua group, and three for the Yorkshire group), respectively; while the *r* and *p* represent the mean in the 18 samples for validation. (**A**) TCONS_00000089 vs. ALDH2; (**B**) TCONS_00000742 vs. PIGC; (**C**) TCONS_00044733 vs. TMEM120A; (**D**) TCONS_00059011 vs. ENSSSCG00000026974.

In the PTG prediction section, we predicted 352 DEPTGs corresponding to 16 DELs based on the expression level. To confirm this result, we performed a regression analysis based on their expression data in another longissimus dorsi muscle dataset. The results revealed a good consistency between two datasets (Figure 6). For example, lincRNA TCON_0000089 had a significantly positive correlation with the aldehyde dehydrogenase 2 family (ALDH2) (Figure 6A), while TCON_00059011 had a significantly negative correlation with ENSSSCG00000026974 (Figure 6D).

## 4. Discussion

In this study, we present the comprehensive identification and analysis of lincRNAs in pig longissimus dorsi muscle, based on published RNA-seq data [2]. We also identified DELs and DEGs that are associated with muscle performance and meat quality based on a designed pipeline. Previous studies have demonstrated that there is a large number of lincRNAs in mammalian genomes, and their exact number may equal or even surpass the number of protein-coding genes [46,47]. So, there are many lincRNAs remaining undiscovered in pig. Here, we identified 354 novel putative lincRNAs, broadening the pig lincRNA annotation. We constructed and improved our new lincRNA identification pipeline through integration with previous published lincRNA identification procedures, and Pfam and BLASTX procedures to reduce false positive and false negative results [23,46,48]. The lincRNAs in our study exhibited some typical features, such as shorter transcript length, longer exon length, fewer exons, and lower expression levels compared with protein-coding genes. Moreover, the correlation between lincRNAs and their PTGs were also validated successfully in another dataset. These results support the effectiveness of our identification and analysis approach, which will aid the identification and functional characterisation of lincRNAs in other tissues or species. Besides, previous studies have proven that lincRNAs exhibit higher tissue-specific properties than protein-coding genes [46,49]. Therefore, we inferred some of the 759 lincRNAs identified in our study may specifically express in longissimus dorsi muscle and exert some functions related to muscle growth and meat quality.

The low expression level and lack of annotation information of lincRNAs makes it more challenging to explore lincRNA functions. Previous studies have demonstrated that lincRNAs can regulate gene expression in *cis* or in *trans* [50,51,52,53]. Hao and Andrea also used protein-coding genes that transcribed nearby (<10 kb) lincRNAs or associated with lincRNAs in terms of expression to study lincRNA functions [48,54]. In this study, we identified a total of 352 DEPTGs of DELs between the YY and WH groups. We explored the lincRNA functions through gene ontology and pathway analysis of their PTGs. We found that some DEPTGs of DELs were involved in the regulation of skeletal muscle tissue development, the glucose metabolic process, and fatty acid metabolism, which are related to muscle growth and meat quality. So, we conclude that the DELs may contribute to the differences between two groups by regulating their PTGs. However, the mechanisms by which DELs regulate their PTGs and further affect the YY and WH pig production performance are still unclear, and deserve further studies.

In order to clarify the regulation relationship between lincRNAs and their PTGs, we summarized their expression status between two groups. We found that 14 of 16 DELs can positively regulate the majority of their PTGs, and only two of 16 DELs can negatively regulate the majority of their PTGs. This result indicated that lincRNAs can regulate PTGs in different ways. We speculated that most DELs can positively regulate their PTG expression and then contribute to the growth and meat quality differences between two groups. In a previous study, Kevin et al. concluded that there are four archetypes (signals, decoys, guides, and scaffolds) of molecular functions that lincRNAs execute, and since lincRNAs belong to different archetype, they have distinctive mechanisms to regulate their PTGs [55]. Besides, many lincRNAs could execute their functions with a combinatorial archetype, such as HOTAIR [56] and COLDAIR [57]. So, we inferred that lincRNAs may regulate their PTGs in certain ways only when those lincRNAs can be classed in a specific archetype.

In this study, we found both the lincRNA TCONS_00006963 and its PTG ANKRD2 (Ankyrin repeat domain protein 2) were significantly upregulated in the WH group compared with the YY group. Previous studies have proven that the upregulation of ANKRD2 can impair myogenic differentiation potential and inhibit myoblast differentiation [58,59], so we inferred that high expression of TCONS_00006963 may upregulate the expression of ANKRD2 in longissimus dorsi muscle in the WH group, and then contribute to a slower growth compared with the YY group. LincRNA TCONS_00000742 was downregulated in the WH group compared with the YY group, and its PTG SLC16A3 (Solute carrier family 16 member 3) exhibited a consistent expression status like TCONS_00000742 between the two groups. In the previous study, SLC16A3 was demonstrated to favour lactate efflux over influx of the cell [60,61]. Therefore, we conjectured that high expression of TCONS_00000742 can upregulate SLC16A3 expression in the YY group, and then increase the lactate content in longissimus dorsi muscle, which may further lead to a lower muscle pH and worse meat quality [62]. Besides, Pilegaard et al. has found that SLC16A3 was more predominantly expressed in muscles rich in fast-twitch (type II) fibres than in muscles rich in slow-twitch (type I) fibres [63], and Choe et al. has proved that muscles with high glycogen and lactate content were composed of significantly higher fiber type IIB and lower fiber type I, and tended to show rapid postmortem glycolysis, paler surface colour, higher drip loss, and high extents of protein denaturation [64]. Considering the poor meat quality of the YY group, we speculated that the YY pigs may have higher fiber type IIB content in longissimus dorsi muscle than WH pigs. Moreover, we found that both lincRNA TCONS_00061360 and its PTG ACACB (Acetyl-CoA carboxylase beta) were upregulated in the WH group compared with the YY group. Acetyl-coenzyme A carboxylase beta (ACACB) is an essential regulator of the fatty acid oxidation pathway [65]. Abu-Elheiga et al. has reported that ACACB knockout mice were protected against obesity and diabetes induced by high fat [66], which may mean that ACACB can promote lipid synthesis or deposit. So, we inferred that the higher expression of TCONS_00061360 in the WH group may positively regulate ACACB expression, and then contribute a higher intramuscular fat content in longissimus dorsi muscle of the WH pigs.

In summary, we performed identification and characterisation of a number of novel lincRNAs in longissimus dorsi muscle of pig. We found a dominant mechanism in which lincRNAs can regulate most of their PTGs and further contribute to performance differences between the WH and YY pigs. Although we found a list of lincRNAs that may lead to growth or meat quality differences between two groups, we couldn’t carry out further functional experiments because of the unavailability of original samples. The mechanisms by which lincRNAs exert to regulate their PTGs are still unclear and deserve further researches. Nevertheless, our study provides novel insights into the discovery and annotation of muscle growth-related and meat quality-related lincRNAs in pig. These lincRNAs, especially DELs with PTGs differentially expressed between two groups, represent ideal candidates for further studies about genes involved in muscle growth-related and meat quality-related processes.

## Figures and Tables

**Table 1:** Summary of data from RNA-seq for the Yorkshire and Wannanhua pigs.

Sample	Accession Number	Raw Reads	Clean Reads	Mapped Reads	Mapping Ratio
WH03	SRR2919657	64,584,126	61,821,920	49,030,410	79.3%
WH05	SRR2919658	64,327,738	61,484,062	48,584,612	79.0%
WH12	SRR2919659	59,244,502	56,584,368	43,757,304	77.3%
YY01	SRR2919660	57,969,134	55,324,162	41,665,620	75.3%
YY02	SRR2919661	68,254,168	64,955,554	49,480,266	76.2%
YY06	SRR2919662	72,298,142	68,884,390	50,825,220	73.8%

**Table 2:** Summary of differentially expressed lincRNAs (DELs) and their differentially expressed potential target genes (DEPTGs).

DELs	Number			DELs	Number		
	DEPTGs	UpRegulated PTGs	DownRegulated PTGs		DEPTGs	UpRegulated PTGs	DownRegulated PTGs
TCONS_00000089	41	34	7	TCONS_00021915	71	27	44
TCONS_00000134	4	4	0	TCONS_00034774	4	4	0
TCONS_00000742	53	37	16	TCONS_00044733	80	76	4
TCONS_00005771	7	7	0	TCONS_00056305	5	5	0
TCONS_00006963	31	26	5	TCONS_00059011	14	3	11
TCONS_00007946	46	42	4	TCONS_00061360	110	79	31
TCONS_00013076	3	2	1	TCONS_00061633	13	13	0
TCONS_00021480	12	11	1	TCONS_00064940	11	10	1

## Supplementary Materials

The following are available online at www.mdpi.com/2073-4425/8/8/203/s1. Table S1: Information of all lincRNAs in this study. Table S2: DELs between the WH group and the YY group. Table S3: Gene ontology and pathway analysis of DEGs. Table S4: Gene ontology and pathway analysis of PTGs of DELs. Table S5: Gene ontology and pathway analysis of differentially expressed PTGs(DEPTGs) of DELs. Table S6: Differentially expressed PTGs(DEPTGs) of differentially expressed lincRNAs (DELs).
